# Patients with hematological malignancies admitted to intensive care units: new challenges for the intensivist

**DOI:** 10.5935/0103-507X.20150040

**Published:** 2015

**Authors:** Viviane Bogado Leite Torres, Marcio Soares

**Affiliations:** 1 Postgraduate Program in Internal Medicine, Universidade Federal do Rio de Janeiro (RJ), Rio de Janeiro, Brazil.; 2 Postgraduate Program in Oncology, Instituto Nacional de Câncer, Rio de Janeiro (RJ), Brazil.; 3 Department of Intensive Care Medicine, Instituto D’Or de Pesquisa e Ensino, Rio de Janeiro (RJ), Brazil.

Advances in treatment of cancer patients and improved understanding of pathophysiological mechanisms behind malignant diseases contribute to increased survival and, consequently, increasing needs of intensive care support for this population.^(1)^ It should be highlighted that ‘cancer’ is a name generically given to a widely heterogeneous group of diseases; in comparison to solid tumors, hematological neoplasms show a number peculiar features. Among the most relevant, it should be emphasized the urgency of starting anti-cancer therapy, as often required in high-grade hematological neoplasms as acute leukemia and aggressive lymphomas. Specific research on this subgroup is warranted, considering the potential prognostic impact of the underlying neoplasm behavior and center-specific features (such as volume of cases, availability of anti-cancer agents and specific diagnosis techniques).^(2)^

In the past two decades, intensive care units (ICU) increasingly played a relevant role, both treating infective intercurrences and severe complications related to the cancer itself and its therapy; and preventive admissions of high-risk patients undergoing chemotherapy.^(3)^ Currently, refusing ICU admissions based only on the type of hematological cancer is no longer justifiable. Therefore, the intensive care specialty faces new challenges represented by severely ill patients with malignant underlying diseases requiring, in addition to traditional intensive care, progressively more specific knowledge on oncology.^(4)^

These new and progressive challenges require the intensivist to be capable of offering both the best clinical care and appropriate advice for patient and family members regarding prognosis, therapeutic options and preferences. Therefore, some behavioral changes are required, particularly regarding improved cooperation between intensivists and oncologists/hematologists. In addition to influencing the clinical practice and decision making on anti-cancer therapy, this interaction may contribute to appropriately select patients who may better benefit from intensive care.^(4)^ A suitable example of such cooperation is giving urgent intravenous chemotherapy to hematological patients during their ICU stay. This cooperation has been shown feasible, adding a positive impact on selected patients’ prognosis, including for those with highly severe diseases.^(3,5)^

Some independent aspects associated to poor prognosis in severely ill hematological cancer patients have been identified, such as the need of invasive respiratory support, more organ dysfunctions, poor performance status and neoplasm organ infiltrations.^(6,7)^ Now, the challenge is to evaluate if these findings translate into bedside benefits in different scenarios.^(8)^ So far, most of the studies assessing this population outcomes in Brazilian ICUs have included solid tumors, rendering difficult interpreting the results.^(9)^

In this issue of RBTI, Barreto et al. report on the two-year assessment of 157 hematological disease adult patients admitted to a general ICU in a Brazilian university hospital.^(10)^ Although conducted in one single center, this study adds relevant information on the scenario of hematological patients in Brazilian ICUs. The authors observed a high prevalence of cancer, 81.6% of this hematological patients’ sample. This translated into one out every six ICU admissions in this timeframe. The reported ICU and hospital mortality rates were 47.8% and 73.2%, respectively. Multiple factors may have contributed to such high rates. Among them, the disease severity upon ICU admission, assessed by SAPS 3 score, was shown to be an independent mortality predictor. These findings highlight the results of recent studies stressing the relevance of early intensive care in severely ill patients. Expert recommendations for widening criteria for admitting hematological cancer patients to the ICU and full intensive care within the first days should be aligned with identifying early stage of critical diseases. Ideally, before organ dysfunctions are installed.^(11)^ Thus, a possible intervention target is apparent, particularly in Brazil, where the access to intensive care is jeopardized by system ineffectiveness and/or shortness of intensive care beds.^(12)^ Recently a European study including hematological cancer patients in 17 ICUs located in France and Belgium identified that ICU admission within the first 24 hours after hospital admission is associated to better survival rates in comparison to the *a priori *anticipated. The reported hospital mortality was 39%, and both cancer disease control and health-related quality of life following discharge were considered satisfactory, suggesting that appropriate cost-benefit ratio was achieved.^(7)^

Respiratory failure is known to be the main cause leading hematological patients to intensive care admission; another relevant contribution by Barreto et al.^(10)^ regards the encouraging hospital survival rates found in patients undergoing noninvasive mechanic ventilation (NIMV). These rates were similar to those found in patients requiring no respiratory support at all. Yet in patients failing to NIMV, the mortality rate was high, even above the rate observed in patients whose first respiratory support option was invasive mechanic ventilation. These results confirm previous results^(13,14)^ supporting both decisions for electing invasive respiratory support in selected patients and the importance of early identification of NIMV failure associated features. In the study by Barreto et al. the subgroup failing to NIMV had more severe respiratory dysfunction and increased use of hemodynamic support within the first 24 hours following ICU admission; this agrees with the literature.^(15)^ According to the current knowledge, upon identification of these features, invasive respiratory support would be the preferable starting strategy.

The study by Barreto et al.^(10)^ reports on relevant information about features of hematological diseases in severely ill patients. However, additional research is warranted to better understand the profile of this population of patients, increasingly admitted to Brazilian ICUs. New data on long-term mortality, health-related quality of life following ICU discharge and characterization of possibly outcome-related ICU issues are necessary for better assessing the care provided to these patients.
